# Differential Tractography and Correlation Tractography Findings on Patients With Mild Traumatic Brain Injury: A Pilot Study

**DOI:** 10.3389/fnhum.2022.751902

**Published:** 2022-01-27

**Authors:** Meng-Jun Li, Fang-Cheng Yeh, Si-Hong Huang, Chu-Xin Huang, Huiting Zhang, Jun Liu

**Affiliations:** ^1^Department of Radiology, The Second Xiangya Hospital of Central South University, Changsha, China; ^2^Department of Neurological Surgery, University of Pittsburgh School of Medicine, Pittsburgh, PA, United States; ^3^Department of Bioengineering, University of Pittsburgh School of Medicine, Pittsburgh, PA, United States; ^4^MR Scientific Marketing, Siemens Healthcare Ltd., Wuhan, China

**Keywords:** mild traumatic brain injury, differential tractography, correlation tractography, fiber tracts, cognitive function, mild traumatic brain injury (mTBI)

## Abstract

Differential tractography and correlation tractography are new tractography modalities to study neuronal changes in brain diseases, but their performances in detecting neuronal injuries are yet to be investigated in patients with mild traumatic brain injury (mTBI). Here we investigated the white matter injury in mTBI patients using differential and correlation tractography. The diffusion MRI was acquired at 33 mTBI patients and 31 health controls. 7 of the mTBI patients had one-year follow-up scans, and differential tractography was used to evaluate injured fiber bundles on these 7 patients. All subjects were evaluated using digital symbol substitution test (DSST) and trail making test A (TMT-A), and the correlation tractography was performed to explore the exact pathways related to the cognitive performance. Our results showed that differential tractography revealed neuronal changes in the corpus callosum in all 7 follow-up mTBI patients with FDR between 0.007 and 0.17. Further, the correlation tractography showed that the splenium of the corpus callosum, combined with the right superior longitudinal fasciculus and right cingulum, were correlated with DSST (FDR = 0.001669) in the acute mTBI patients. The cognitive impairment findings in the acute stage and the longitudinal findings in the corpus callosum in the chronic stage of mTBI patients suggest that differential tractography and correlation tractography are valuable tools in the diagnostic and prognostic evaluation of neuronal injuries in mTBI patients.

## Highlights

- Differential tractography and correlation tractography were adopted to identify neuronal injury in mild traumatic brain injury patients.

## Introduction

Mild traumatic brain injury (mTBI) is the most common type of traumatic brain injury (TBI), accounting for 75–90% of TBI cases (Mondello et al., 2014; Galgano et al., 2017). Although most mTBI patients are asymptomatic within several days or weeks after injury, 10–30% patients show long-term symptoms, including somatic complaints (headache, dizziness, fatigue, sleep disturbance), behavioral and emotional disorders (anxiety, feeling depressed or tearful, being irritable, reduced self control, loss of initiative and motivation) and subjective cognitive impairment (slowness, memory deficits, concentration difficulties) (Messé et al., 2011).

The diffusion tensor imaging (DTI), which is sensitive to white matter axonal injuries based on Gaussian diffusion, has been used to evaluate fiber tracts damage following mTBI (Wallace et al., 2018; Zhu et al., 2019). These Gaussian diffusion based parameters, including fractional anisotropy (FA), radial diffusivity (RD) and mean diffusivity (MD), which did not consider the non-Gaussian diffusion behavior of *in vivo* water diffusion within fiber tracts, were measured to reflect the changes of white matter and myelin microstructure after injury. Higher FA, combined with the higher RD and MD, were identified in the mTBI patients when the injury was onset within days, indicating neuronal swelling or cytotoxic edema in the acute stage of the mTBI. Then, the FA decreased to normal during the semi-acute stage in most of the mTBI patients, accompanied with the improvement of post-injury symptoms. While, the athletes with more than one mTBI, combined with the children and young adult populations, had longer increased FA. Furthermore, the patients with persistent post-injury symptoms also had a prolonged period of increased FA. In addition, these patients with persistent post-injury symptoms had lower FA in the chronic stage when compared to healthy controls, and the white matter integrity was correlated with the post-injury symptoms (Chong and Schwedt, 2018). These findings showed loss of myelin and degenerative changes in the mTBI patients with persistent symptoms and demonstrated that the DTI was sensitive in the detection of group-level abnormalities and in the assessment of chronic fiber tract changes.

However, the DTI findings at the group-level may not be applicable for mTBI patient at the individual-level due to the heterogeneity within mTBI patients, which comes from the cause of mTBI (adult civilian, traffic accident, military and sport-related mTBI) and injury position (frontal, temporal, parietal and other position). Moreover, discrepant DTI findings in white matter diffusion metrics were reported across mTBI studies (Asken et al., 2018). For example, high anisotropic diffusion (AD)/low radial diffusivity (RD) in the genu of the corpus callosum was consistently observed in both the replication and original mTBI cohorts (Ling et al., 2012), while another study revealed that acute mTBI was not associated with DTI abnormalities with tract-based spatial statistics in a large and carefully screened sample (Ilvesmäki et al., 2014). The possible reasons for discrepant DTI findings might be control group variability, the mTBI heterogeneity, the analysis techniques, the methods in which the regional differences were reported, and the persistent functional disturbances (Asken et al., 2018). Meanwhile, the complex microstructure, such as crossing fibers, kiss fibers and etc., cannot be resolved by the traditional DTI-based fiber tracking, as well as the exact originations and destinations of fibers (Fernandez-Miranda et al., 2012). Furthermore, the indices in DTI are susceptible to partial volume effects (Oouchi et al., 2007). Additionally, the influence of free-water cannot be removed from the traditional DTI data (Tang et al., 2019).

Recently, differential tractography (Yeh et al., 2019b) and correlation tractography (Yeh et al., 2016a) have been proposed as new tractography modalities to study the white matter tracts. Specifically, differential tractography utilizes repeat MRI scans of the same subjects at different time points to map the exact segment of fiber pathways with neuronal injury in the longitudinal studies. As a quantitative and objective method, it has metrics of monitoring neuronal injury in a single subject without considering inter-subject and group variability, thus allowing for diagnostic and prognostic evaluation of brain diseases at individual-level. Compared to the conventional tractography that maps all the existing pathways, differential tractography could track the precise segments of pathways showing longitudinal changes. Therefore, it has been used to detect neuronal injury on multiple sclerosis, Huntington’s disease, amyotrophic lateral sclerosis, and epileptic patients (Yeh et al., 2019b). The affected pathways shown by differential tractography matched well with the unique clinical symptoms of the patients, and the false discovery rate of the findings could be estimated using a sham setting to provide a reliability measurement. On the other hand, correlation tractography tracks the precise segment of pathways correlated neuropsychological scores in a group of subjects. The results could inform the structure-function relation and explain how white matter affects cognitive functions by disrupting brain circuits. Conventional tractography analysis, which mapped the connections between different brain areas through fiber tracking, has been questioned and its limitations may render track-specific analysis inconclusive. The correlation tractography is more accurate in reflecting the structure and density of the white matter tracts considering crossing fibers and partial volume effects. Hence, it has been applied to map brain connections and correlate findings in neuropsychological disorders (Yeh et al., 2013a; Delaparte et al., 2017; Wang et al., 2019) and neurodegenerative diseases (Mojtahed Zadeh et al., 2018). The differential tractography and correlation tractography have been used to study neuronal changes in multiple brain diseases, but their performances in detecting neuronal injury are yet to be investigated in mTBI patients.

In this study, we aimed to investigate the white matter injury in single-subjects through the fiber tract comparisons between the acute and the follow-up mTBI patients with the differential tractography. Firstly, we performed group comparisons for the acute mTBI patients and the healthy controls with cross sectional differential tractography. Then, the fiber tracts 1 year after injury onset were compared to the white matter of the acute stages from the same patients through the longitudinal differential tractography to evaluate neuronal alterations in single-subject. After that, correlation tractography was performed to find out the exact fiber tracts that correlated with the cognitive behavior. To our knowledge, this is the first study to evaluate trajectory alterations of brain pathways injured from mTBI with differential tractography and correlation tractography, and it can better our understanding in the circuit pathology behind mTBI and the correlation with cognitive manifestations.

## Methods

### Participants

All of the mTBI patients were enrolled from September 2019 to December 2020. The mTBI patients were pre-screened prior to scanning to rule out any contraindications to MRI. Inclusion criteria for mTBI patients were based on the World Health Organization’s Collaborating Center for Neurotrauma Task Force (Holm et al., 2005). Exclusion criteria for mTBI patients were as follows: (1) a history of previous brain injury, (2) penetrating craniocerebral injury and/or presence of a skull fracture, (3) the mTBI due to other injuries (e.g., systemic injuries, facial injuries, or spinal cord injury), (4) a history of neurological disease, long-standing psychiatric condition or other problems (e.g., psychological trauma, language barrier), (5) coexisting medical conditions and/or drug abuse (e.g., alcohol abuse, administration of sedatives), (6) structural abnormality on neuroimaging (computed tomography and MRI).

Healthy controls were enrolled from the healthy check-up at the same term in the Second Xiangya Hospital. They were pre-screened before scanning to rule out any contraindications to MRI, neurological impairment and psychiatric disorders. Finally, a total of 33 acute mTBI patients (Gender: 13 males and 20 females; Mean age: 36.6 ± 11.5 years; Age range: 18–59 years) and 31 healthy controls (Gender: 13 males and 18 females; Mean age: 38.4 ± 8.3 years; Age range: 23–51 years) were recruited in this study, and 7 mTBI patients came back for check after one year. Approval was granted by the Ethics Committee of the Second Xiangya Hospital of Central South University (approval No. 086) on February 9 2019. Written informed consent was obtained from all participants before testing.

### MRI Data Acquisition and Preprocessing

MRI data were acquired from mTBI patients at 7 days and 1 year after brain injury on a 3.0T MRI scanner (MAGNETOM Skyra, Siemens Healthcare, Erlangen, Germany) with a 32-channel head coil. A head stabilizer was used to reduce the head motion. A 2D echo-planar imaging sequence was used to obtain the diffusion-weighted MRI data, and the parameters were as follows: *b*-values: 0, 1000, 2000 s/mm^2^, 10 diffusion directions for zero b value and 64 diffusions directions at each non-zero b values, echo time (TE): 92 ms, repetition time (TR): 5400 ms, voxel size: 2mm × 2mm × 3mm, field of view (FOV): 224mm × 224mm. To achieve high resolution anatomical comparisons, a T1 magnetization prepared rapid gradient echo (MPRAGE) sequence was performed with the following parameters: TR: 2400 ms, TE: 2.7 ms, flip angle: 8°, and voxel size: 1mm × 1mm × 1mm. After data acquisition, the movement correction and eddy current correction of the diffusion data were pre-processed using the FSL5.0.9. Then, the pre-processed diffusion data were analyzed by DSI studio^1^. Generalized q-sampling imaging (GQI) (Yeh et al., 2010) was used to reconstruct the spin distribution function (SDF) map. The cerebral lesions and micro-bleeds were inspected independently by the two authors (CXH and JL) with over ten years’ experience in neuroimaging. Any disagreements between these them were resolved by consensus.

### Clinical Assessments

Clinical assessments were performed for all the participants after MR imaging. To avoid multiple testing issues, two tests were selected for cognitive assessment: (1) The Digital Symbol Substitution Test (DSST). The participants’ processing speed, sustained attention and working memory were assessed by the DSST test (Wechsler, 1997; Qin et al., 2017). Participants were shown 9 numbers and corresponding symbols and then instructed to match them in two minutes. The total score they got was the number of the correctly matched symbols. More correctly matched symbols indicated better performance in the assessment; (2) The Trail Making Test A (TMT-A)(Reitan and Wolfson, 1993). TMT-A was administered as a baseline measure of motor and visual search speed (Sánchez-Cubillo et al., 2009; Misdraji and Gass, 2010). Each participant was instructed to draw lines connecting numbers consecutively from one to twenty-five as quickly as possible. The score was the time (in seconds) required to complete the task. Shorter time indicated better performance. These two tests have been widely used in neuropsychological assessments as indicators of cognitive processing speed and executive functioning.

### Group Differential Tractography

Group average templates were constructed by averaging the SDF for the mTBI and healthy control groups in DSI Studio. The average templates (mTBI and healthy controls) were used to examine the representative brain connections and their differences. To generate a group average SDF, the diffusion data from each participant were reconstructed in the MNI space through DSI Studio using q-space diffeomorphic reconstruction to obtain the SDF with the default settings where the diffusion sampling length ratio was 1.25 and the output resolution was 2 mm. Then the SDF from all the participants were averaged into a population average template to produce the group average SDF in DSI Studio. Finally, the averaged group SDF-values, calibrated by free water diffusion in the ventricles, were compared between the mTBI and the healthy control group with the default Human Connectome Project (HCP)-1021 as the template, which was reconstructed from a total of 1021 subjects’ diffusion MRI data from the HCP (Yeh and Tseng, 2011; Yeh et al., 2018).

The procedures to confirm the axonal injuries were as follows. Firstly, the differential tractogram was obtained by placing a total of 100,000 seeding points in the white matter. The angular threshold was 60°. The step size was 1 mm (Yeh et al., 2019b). The anisotropy threshold was determined by the quality check under the whole brain fiber tracking. To evaluate the potential changes of the fiber tracts, the differential tractography was performed with different quantitative anisotropy (QA) change thresholds (10, 20, and 30%) and fiber length thresholds (20, 30, and 40 mm), the low QA change thresholds and fiber length thresholds are more sensitive for early demyelination and the high QA change thresholds and fiber length thresholds are more specific for axonal loss (Yeh et al., 2019b). Tracks in lengths shorter than the length thresholds and the tracks in changes less than the QA change thresholds were discarded. The increase anisotropy and the decrease anisotropy were calculated according to the different length thresholds and the QA change thresholds, respectively. Finally, the false discovery rate (FDR) was calculated as increase anisotropy by decrease anisotropy. The FDR ranged from 0.05 to 0.2 indicates potential axonal loss, and is confirmative of axonal loss when lower than 0.05. The FDR has already considered multiple comparisons, and there is no risk of inflating the significance.

### Individual Differential Tractography

The individual differential tractography was performed between the scan of the acute stage and the chronic stage from the same mTBI patients. The SDF was then acquired by GQI. The individual differential tractogram was obtained using the same method as group differential tractography tractogram. At the same time, the differential tractography was also performed with different FA change thresholds (5, 10, and 15%) and fiber length thresholds (10, 20, 30, and 40 mm). The FDR has already considered multiple comparisons, and there is no risk of inflating the significance.

### Correlation Tractography

Diffusion MRI connectometry enabled us to further investigate the QA of specific pathways associated with the DSST and TMT-A scores in mTBI patients. Firstly, mTBI connectometry database was created based on the q-space diffeomorphic reconstruction, and post-reconstruction quality check was performed. Then, a non-parametric Spearman partial correlation was used to derive the correlation, and the effect of sex, age, and education was removed using a multiple regression model. To map the different levels of correlation between the tracks and the DSST and TMT-A, different T thresholds (2, 2.5, 3.0, and 3.5) were used to study the correlation at different significance levels using a deterministic fiber tracking algorithm, the high T thresholds will map tracks with a stronger correlation effect, whereas lower T thresholds will map tracks with a weak correlation (Yeh et al., 2016a). The QA-values were normalized. The tracks were filtered by topology-informed pruning with 4 iteration(s) (Yeh et al., 2019a). A length threshold of 40 voxels distance was used to select tracks. To estimate the false discovery rate, a total of 4000 randomized permutations were applied to the group label to obtain the null distribution of the track length (Yeh et al., 2016a). The FDR less than 0.05 indicated a highly confirmative association between the specific fiber tracts and cognitive scores. After the group connectometry analysis, the network property analysis was performed based on the correlations. A deterministic fiber tracking algorithm was applied to generate whole-brain tractography based on the default setting of anisotropy threshold, angular threshold, and step size in the DSI Studio. A total of 1,000,000 tracts were calculated for the connectivity matrix based on an automated anatomical labeling atlas (AAL), which is a digital human brain structure atlas drawn by Montreal Neurological Institute (MNI) based on the MNI single-subject T1-weighted structure image template (Rolls et al., 2020). After that, the clustering co-efficiency average, network characteristic path, small worldness, and global efficiency were calculated in the DSI Studio followed the implementation of the brain connectivity toolbox^2^ (Bullmore and Sporns, 2009; Rubinov and Sporns, 2010). Then, the significance fiber tracts found in the correlation analysis were filtered in the whole brain tracks, and the differences of values were calculated for clustering co-efficiency average, network characteristic path, small worldness, and global efficiency.

### Statistical Analysis

Firstly, the normality distribution of continuous variables in mTBI group and healthy controls was tested by the Shapiro–Wilk *W*-test. Then, the independent two-sample *t*-test and the Mann–Whitney test were applied to compare group differences for the data normality distribution and data non-normality distribution, respectively. Chi-square analyses were used to assess the differences of categorical variables. Paired-sample *t*-test was used to assess the difference of DSST and TMT-A in the chronic stage and in the acute stage. *P* < 0.05 was considered to indicate a significant difference.

## Results

### Demographic and Clinical Characteristics of Mild Traumatic Brain Injury Patients and Healthy Controls

All the recruited subjects were confirmed to have minimal head movement during scanning and included for analysis. No significant differences were observed between the mTBI and healthy control for mean age (*P* = *0.489, t* = *−0.696*), education level (*P* = *0.706, U* = *484.000*) and frequency of gender (*P* = *0.836*χ^2^ = *0.043*). The demographic data and clinical characteristics were shown in Table 1.

**TABLE 1:** Demographic and Clinical Assessment in mild traumatic brain injury (mTBI) Patients and Healthy Controls.

Demographic Characteristics	mTBI Patients (*n* = 33)	Health Control (*n* = 31)	Statistical Significance
Age (yr) *	36.6 ± 11.5	38.4 ± 8.3	***P* = *0.489 t* = *−0.696***
Education level (yr) *	12.8 ± 3.5	13.2 ± 3.6	***P* = *0.706 U* = *484.000***
Female^#^	20(60.6%)	18(58.1%)	***P* = *0.836*χ^2^ = *0.043***
**Mechanism of Injury** ^#^			
Motor Vehicle Accident	11(33.3%)	–	
Assault and hit	9(27.3%)	–	
Fall	7(21.2%)	–	
Other	6(18.2%)	–	
**Cognitive Assessment** *			
DSST (n)	45.3 ± 14.3	54.0 ± 16.8	***P* = *0.032 t* = *−3.002***
TMT-A (s)	56.1 ± 31.7	46.4 ± 19.9	***P* = *0.248 U* = *412.000***

*Data are expressed as mean ± SD (*) and number (percentage) (#). Age and DSST were analyzed by independent two-sample t-test. Education level and TMT-A were analyzed by Mann–Whitney test. Female was analyzed by Chi-square test. DSST, Digital Symbol Substitution Test; mTBI, mild traumatic brain injury; TMT-A, Trail Making Test A. Bold values represent statistical significance at P < 0.05.

The healthy controls performed better than the mTBI patients in the cognitive assessments. For the TMT-A tests, more time was spent for mTBI patients than the healthy controls. However, no significant differences between the two groups (*P* = *0.248, U* = *412.000*) were observed. Significant difference was observed for the DSST score between mTBI patients and the healthy controls (*P* = *0.032, t* = *−3.002)*. The mTBI patients matched fewer correct symbols in DSST test than the healthy controls. The TMT-A test, combined with the DSST, indicated a cognitive impairment in the acute mTBI patients. A detailed statistical analysis was summarized in the Table 1.

### Group Differential Tractography

**FIGURE 1:**
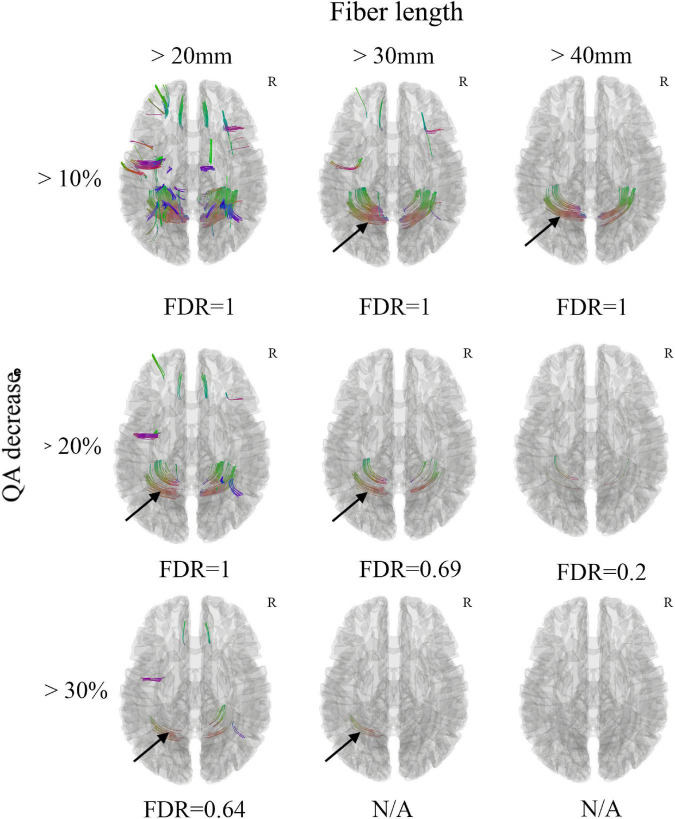
Differential tractography of acute mild traumatic brain injury (mTBI) patients in comparison with health controls through group average SDF template. Decreased QA in the cerebellum fiber tracts was observed (black arrow), while the FDR was larger than 0.20, which means that the QA decrease in these cerebellum fiber tracts was not significant. Furthermore, these cerebellum tracks were located in the bottom slicers, it was suggested to remove these findings according to the differential tractography tutorials. The FDR would be non-available when there are too few/no QA findings. N/A, non-available; R, right hemisphere.

White matter with significant difference was not found between the acute mTBI patients and the healthy controls from the group average SDF (Figure 1). When the differential tracking thresholds ranged from 10, 20, and 30% and the min fiber lengths ranged from 20mm, 30mm and 40mm, decreased QA in the cerebellum fiber tracts was identified. In the meantime, the FDR was larger than 0.20, which means that the QA decrease in these cerebellum fiber tracts was not significant. Furthermore, since these cerebellum tracks were located in the bottom slicers, it was suggested to remove these findings according to the differential tractography tutorials. The FDR would be non-available when there are no increase QA findings.

### Individual Differential Tractography

**FIGURE 2:**
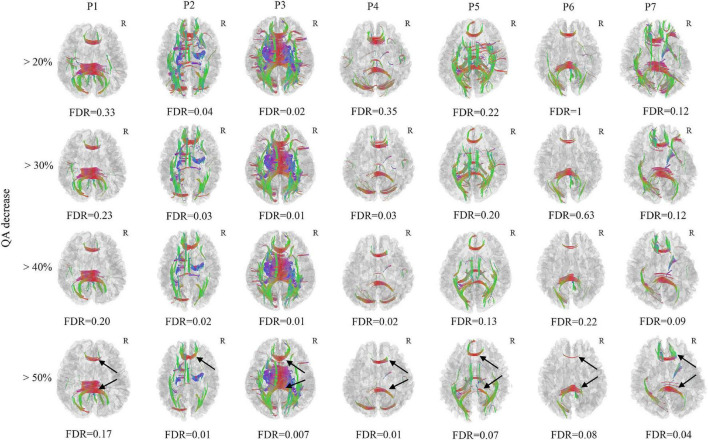
Differential tractography of 7 chronic mild traumatic brain injury (mTBI) patients in comparison with the same 7 mTBI patients at acute stage in single-subject. Axonal loss of corpus callosum was confirmed in Patient 2, Patient 3, Patient 4, and Patient 7 (FDR < 0.05) when the anisotropy decreased larger than 50% and the min fiber length was 30mm, it was also observed in Patient 1, Patient 5, and Patient 6 (FDR ranged from 0.05–0.20). The axonal loss of corpus callosum was mostly located in the splenium and genu of the corpus callosum (black arrow). For patient 3, who developed depression, a much larger number of axonal loss than other asymptomatic patients was observed. R, right hemisphere.

Only 7 mTBI patients completed the follow-up diffusion-weighted imaging scans and cognitive performance tests one year after injury due to the emerging COVID-19. All 7 patients recovered from the mTBI in the cognitive impairment and performed significantly better in the DSST (*P* = *0.048, t* = *−2.469)* and TMT-A (*P* = *0.017, t* = *3.270)* test (Table 2). However, Patient 3 developed depression after onset of mTBI. Axonal loss of corpus callosum was observed in Patient 2, Patient 3, Patient 4 and Patient 7 (FDR < 0.05) when the anisotropy decreased more than 50% and the min fiber length was 30 mm (Figure 2). It was also observed in Patient 1, Patient 5, and Patient 6 (FDR ranging from 0.05 to 0.20). For patient 3, who developed depression, a much greater amount of axonal loss in the corpus callosum than other asymptomatic patients was observed.

**TABLE 2:** The digital symbol substitution test (DSST) and trail making test A (TMT-A) results of 7 follow-up chronic mTBI patients.

	age	sex	DSST(n) A/C	TMT-A(s) A/C
P1	18	M	60/62	51.5/36.4
P2	36	F	43/72	70.4/37.8
P3	31	F	48/48	41.5/35.0
P4	26	F	53/56	58.5/46.8
P5	55	M	57/69	47.8/35.4
P6	56	M	17/40	119.9/63.4
P7	38	F	35/40	74.4/58.4
Average			44.7 ± 14.9/55.3 ± 13.1	66.3 ± 26.4/44.7 ± 11.8
***P***			***P* = *0.048***	***P* = *0.017***
***t***			***t* = *−2.469***	***t* = *3.270***

*The DSST and TMT-A in the chronic stage were compared to the acute stage by paired-sample t-test. A/C, Acute/chronic; DSST, Digital Symbol Substitution Test; mTBI, mild traumatic brain injury; TMT-A, Trail Making Test A. Bold values represent statistical significance at P < 0.05.*

**FIGURE 3:**
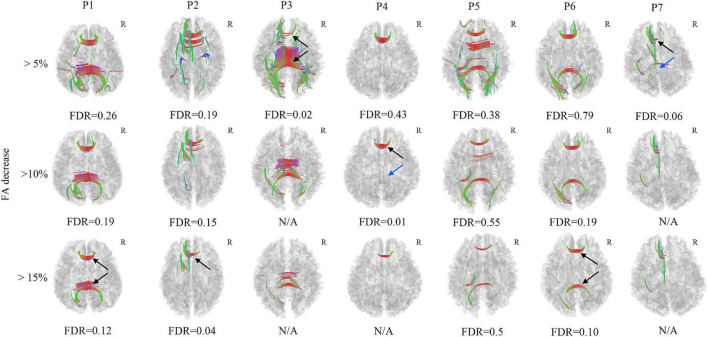
Axonal loss of corpus callosum was confirmed in Patient 2, Patient 3, and Patient 4 (black arrow), the FDR was less than 0.05, it was also observed in Patient 1, Patient 6, and Patient 7, the FDR ranged from 0.05 to 0.20 (black arrow). The axonal loss of corpus callosum in Patient 5 was not significant since the FDR was larger than 0.20. The axonal loss of corpus callosum in the splenium was not identified in Patient 4 and Patient 7 (blue arrow). The FDR would be non-available when there are too few/no increase FA findings. N/A, non-available, R, right hemisphere.

In the FA differential tractography, axonal loss of corpus callosum was confirmed in Patient 2, Patient 3, and Patient 4 (FDR < 0.05). It was also observed in Patient 1, Patient 6, and Patient 7 (FDR ranging from 0.05 to 0.20). The axonal loss of corpus callosum in Patient 5 was not significant since the FDR was more than 0.20 (Figure 3). Furthermore, the axonal loss of corpus callosum in the splenium was not identified in Patient 4 and Patient 7. Compared to the FA differential tractography, the QA differential tractography was more sensitive in identifying axonal loss of fiber tracts. The FDR would be non-available when there are too few/no increase FA findings.

### Correlation Tractography

**FIGURE 4:**
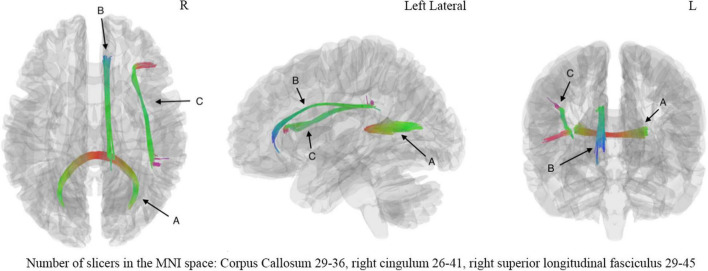
The correlation analysis showed significant correlation (FDR = 0.001669) between digital symbol substitution test (DSST) and fiber bundles including (A) corpus callosum, (B) right cingulum, (C) right superior long fasciculus. The number of slicers of corpus callosum ranged from 29 to 36 in the MNI space, the number of slicers of right cingulum ranged from 26 to 41 in the MNI space, the number of slicers of right superior longitudinal fasciculus ranged from 29 to 45 in the MNI space. L, left hemisphere; R, right hemisphere.

The correlation tractography with a *T*-score of 2.0, 2.5, 3.0, 3.5 was performed in 33 mTBI patients, and the length threshold of 40 voxel distance was applied to select tracks. We found that the splenium of the corpus callosum, combined with the right cingulum and right superior longitudinal fasciculus, showed that QA was positively correlated with DSST at *T*-score of 3.5 (Figure 4), the FDR was 0.001669. No fiber bundles associated with TMT-A test were found in the correlation study. Through the network property analysis for the connectometry, we found that the cluster coefficient declined by 2.69%, the small-worldness by 1.69% and the global efficiency by 2.11%, respectively, combined with an increase of network characteristic path by 3.54% after the significant fiber bundles found in the correlation analysis were filtered.

## Discussion

This study investigated the white matter alterations and cognitive impairment in the mTBI patients with correlation tractography and differential tractography. The cognitive tests confirmed that the mTBI patients had cognitive impairment in the acute stage. At the same time, the correlation tractography demonstrated that the splenium of the corpus callosum, combined with the right superior longitudinal fasciculus and right cingulum, showed that QA was positively correlated with DSST in the acute mTBI patients. In addition, the network property analysis exhibited decrease in clustering co-efficiency average, small-worldness and global efficiency, and increase of network characteristic path after these correlated fiber bundles were removed. These findings indicated that there maybe an injury in these three fiber bundles at the acute stage of mTBI which resulted in the following cognitive impairments. Furthermore, axonal loss in the corpus callosum was identified in 7 follow-up mTBI patients at individual-level using differential tractography, implying that the potential injury in the corpus callosum resulting from the mTBI may cause axonal loss in the follow-up stage. The potential injury in the right superior longitudinal fasciculus and right cingulum may recover in the follow-up stage, accompanied with better cognitive performance in all the 7 follow-up mTBI patients. Our study demonstrated the cognitive impairment in the acute stage of mTBI, and implied potential axonal loss in the corpus callosum at the chronic stage of mTBI with correlation tractography and differential tractography. Larger sample size longitudinal study is needed to confirm these whiter matter changes in the chronic stage. In addition, the relationship between the QA decrease of injured fiber tracts and the clinical manifestations of individual mTBI patients should be examined in future large study. The differential tractography could be used as a quantitative and objective method to detect neuronal injury in individual mTBI patient.

Previous studies have investigated the fiber bundle changes associated with the cognitive function in mTBI patients through DTI. Lower FA values in specific fiber tracts, including the superior longitudinal fasciculus, cingulum, splenium and genu of corpus callosum, were identified correlating with worse cognitive function (Zhu et al., 2019). The cingulum was mainly associated with the memory and general function, the corpus callosum and superior longitudinal fasciculus mainly participated in the executive function, the corpus callosum was also related to the attention and processing speed (Zhang et al., 2019; Zhu et al., 2019). Moreover, decrease of FA in the superior longitudinal fasciculus, cingulum, and corpus callosum was also identified in the Alzheimer’s disease, multiple sclerosis, amyotrophic lateral sclerosis, Parkinson’s disease and other neurodegenerative diseases and correlated with worse cognitive function in these diseases (Hulst et al., 2013; Zhang et al., 2016; Lo Buono et al., 2020; Wang et al., 2020; Chenji et al., 2021). In our study, the specific fiber tracts identified to correlate with the cognitive function in the correlation tractography, was in line with the findings in previous studies. The higher QA in the splenium of the corpus callosum, right superior longitudinal fasciculus and right cingulum, predicted better cognitive performance in the acute mTBI patients. The cognitive impairment identified in the mTBI patients implied potential injury to these three fiber bundles, though no specific fiber tract was found through group comparisons. The further decreased FA and QA in the corpus callosum, identified among the 7 follow-up mTBI patients using differential tractography, implied potential injury of the corpus callosum after mTBI. The FA, which reflected the diffusivity of the water in the fiber bundle, was good for detecting the integrity of the white matter. The decreased FA in the corpus callosum indicated potential loss of myelin and degenerative changes in this fiber bundle. The QA, which measures the density of anisotropic diffusion water in the fiber bundle, was good for quantifying the amount of the diffusing water along the white matter. The decreased QA in the corpus callosum implied potential changes of the diffusion pattern in this fiber tract (Yeh et al., 2016b). Both decrease of the FA and the QA in the corpus callosum implied potential axonal loss in this fiber tract. Interestingly, all the 7 patients achieved significantly better cognitive performance 1 year after injury, which maybe attributed to the repairmen of the longitudinal fasciculus and cingulum after mTBI. Therefore, we cannot find axonal loss in these two fibers using differential tractography.

Compared to the traditional Gaussian diffusion based DTI, GQI is a model-free method that calculate the SDF directly from diffusion MR signals (Yeh et al., 2010). DTI will result in a large variation in the complexity of the biological changes due to its limitation in the restricted diffusion contributed by axonal myelination. The GQI can reconstruct fibers in complex neuroanatomical regions (Panesar et al., 2019; Li et al., 2021a,b) and tumors surrounding edema zones with accuracy (Zhang et al., 2013; Celtikci et al., 2018). Furthermore, the QA, an anisotropy index calculated from the peak orientations on SDF through GQI, is less sensitive to the partial volume effects of crossing fibers. Additionally, the QA-aided tractography has better resolution than FA-aided tractography and has less false fiber tracks in both shell scheme and grid scheme (Yeh et al., 2013b). Moreover, the QA eliminates the free water effect by removing the isotropic diffusion component and includes difference in the density of diffusion spins so that it is more sensitive. In our presented method, the GQI-based differential tractography was more sensitive in the identification of axonal loss. In the FA differential tractography analysis, corpus callosum axonal loss was only confirmed in Patient 2, Patient 3, and Patient 4, while the axonal loss of corpus callosum in Patient 5 was not significant. At the same time, the axonal loss of corpus callosum in the splenium was not identified in Patient 4 and Patient 7. These findings indicated that the GQI-based differential tractography would be a complementary tool for the DTI in the evaluation of the fiber tract microstructure alterations. Moreover, Patient 3, who developed depression, had a much greater amount of QA and FA decrease in the corpus callosum than other asymptomatic patients, prompting that the symptomatic patients may have much more axonal loss in the chronic stage.

It was important for doctors to assess white matter injury at individual-level since the white matter injury in mTBI patients may be present at the microscopic and molecular level undetectable by structure imaging modality (Shin et al., 2017). However, the previous studies focused on the injury at group level and neglected the inter-subject variability, and cannot be used to evaluate white matter injury at individual-level. Recently, more and more studies focused on the development of a predictive modal to find out potential diffusion biomarkers and to detect mTBI patients based on machine learning (Vergara et al., 2017). As a recent prediction modal that can identify mTBI patients by developing information processing speed deficits with 96.7% accuracy through the combination of DTI indices and inflammation cytokines levels, the frontal-subcortical neuronal circuits would be a potential diffusion predictor for processing speed performance in mTBI patients (Bai et al., 2020). Compared to these prediction modals, the differential tractography would be an indispensable complementary tool to evaluate axonal loss for individual mTBI patients. The differential tractography could not only map the exact injured fiber tracts after mTBI, but also quantify the loss of injured white matter in individuals. As a quantitative and objective method to monitor neuronal alterations in single-subject, it is quite important for the diagnosis and prognosis of mTBI, since many functional and metabolic abnormalities in mTBI may be present in the absence of structural damage. This would provide an objective and quantitative index to evaluate the injury and/or repairmen of white matter for individual mTBI patient.

There are several limitations in our study: (1) Although the alterations of fiber tracts in the chronic stage of mTBI patients were found in our study, longitudinal analysis of larger samples is needed confirm these whiter matter alterations. Furthermore, the axonal alterations should be monitored at multiple time points: including 3 months, 6 months, 1 year, and 2 years after injury. It is necessary to evaluate the injury and/or repairmen of fiber tracts in a dynamic process. (2) Due to the limited number of symptomatic patients in this study, the correlation between the QA decrease of injured fiber tracts and the clinical manifestation should be evaluated in larger sample studies, and the objective differential tractography metrics should be found to reflect the axonal loss of white matter and the clinical symptoms at different stages of individual mTBI patients. (3) The causes of mTBI in our study were heterogeneous, including motor vehicle collision, assault, fall, etc., and the locations of the injury were also various. A homogenous study of mTBI, such as the same location or the same cause of injury would minimize the confounding effect. (4) Only the white matter changes were assessed. Multiple modals should be used in future investigations, including cortical thickness, cortical surface area and cortical volume analysis for cerebral cortex, resting state functional connectivity study for dynamic changes in functional networks, arterial spin labeling technology for cerebral blood flow. Graph theory analysis should also be used in the analysis of spatial relations between brain regions at the global and nodal level. A combination of these modals would be helpful in understanding the injury mechanism of mTBI.

## Conclusion

This pilot study demonstrated the cognitive impairment in the acute stage of mTBI. In addition, axonal loss in the corpus callosum was implied at the chronic stage of mTBI patients based on the correlation tractography and differential tractography. These techniques provided a quantitative and objective method to detect neuronal injury in individual mTBI patient, which could be valuable neuroimaging tools to provide clues to the pathophysiological process of white matter alterations in mTBI patients.

## Data Availability Statement

The original contributions presented in the study are included in the article/supplementary material, further inquiries can be directed to the corresponding author/s.

## Ethics Statement

The studies involving human participants were reviewed and approved by the Ethics Committee of the Second Xiangya Hospital of Central South University. The patients/participants provided their written informed consent to participate in this study.

## Author Contributions

M-JL and JL contributed to conception and design of the study. S-HH, C-XH, and JL organized the database. M-JL and F-CY contributed to statistical analysis. M-JL and HZ wrote the first draft of the manuscript. All authors contributed to manuscript revision, read, and approved the submitted version.

## Conflict of Interest

HZ was employed by the company Siemens Healthcare (China). The remaining authors declare that the research was conducted in the absence of any commercial or financial relationships that could be construed as a potential conflict of interest.

## Publisher’s Note

All claims expressed in this article are solely those of the authors and do not necessarily represent those of their affiliated organizations, or those of the publisher, the editors and the reviewers. Any product that may be evaluated in this article, or claim that may be made by its manufacturer, is not guaranteed or endorsed by the publisher.

## Footnotes

1
http://dsi-studio.labsolver.org/

2
https://sites.google.com/site/bctnet/
